# Machine Learning-Based Prediction of Critical Deterioration in the PICU

**DOI:** 10.1097/CCE.0000000000001397

**Published:** 2026-04-22

**Authors:** Sanjiv D. Mehta, Eamonn Tweedy, Victor M. Ruiz, Helen Lingyun Shi, Ryan W. Morgan, Robert M. Sutton, Akira Nishisaki, Fuchiang (Rich) Tsui

**Affiliations:** 1 Division of Critical Care Medicine, Department of Anesthesiology and Critical Care Medicine, The Children’s Hospital of Philadelphia, Philadelphia, PA.; 2 Department of Anesthesiology and Critical Care Medicine, University of Pennsylvania, Philadelphia, PA.; 3 Department of Biomedical and Health Informatics, Children’s Hospital of Philadelphia, Philadelphia, PA.; 4 Tsui Laboratory, Department of Biomedical and Health Informatics, Children’s Hospital of Philadelphia, Philadelphia, PA.; 5 Department of Biostatistics, Epidemiology, and Informatics, University of Pennsylvania, Philadelphia, PA.

**Keywords:** cardiac arrest prevention, clinical deterioration, critical care medicine, machine learning, pediatrics

## Abstract

**BACKGROUND:**

Existing PICU early warning systems lack sufficient accuracy and timeliness for effective preparation. Machine learning approaches may improve prediction of critical deterioration events (CDEs), but their operational utility relative to existing tools remains unclear.

**OBJECTIVES:**

To develop a machine learning model for early detection of CDEs and evaluate operational utility against existing tools using a novel alert burden analysis.

**DERIVATION COHORT:**

PICU admissions (ages 0–24 yr, stay ≥ 24 hr) at a quaternary children’s hospital from 2014 to 2020 (*n* = 12,771 patients; 21,141 admissions). CDEs (6% of patients) included cardiopulmonary resuscitation, extracorporeal membrane oxygenation initiation, dilute epinephrine administration, or unplanned intubation.

**VALIDATION COHORT:**

Temporally distinct PICU admissions from 2021 to 2022 (*n* = 5144 patients; 6929 admissions; 6% CDE rate).

**PREDICTION MODEL:**

An ensemble of extreme gradient-boosted models (PICU Warning INdex [P-WIN]) trained to predict CDEs at 1–12-hour horizons using 550 features derived from demographics, medications, laboratory results, and vital signs.

**RESULTS:**

P-WIN demonstrated excellent discrimination at 2-hour (area under the receiver operating characteristic curve [AUROC], 0.95 [95% CI, 0.94–0.96] and area under the precision-recall curve [AUPRC], 0.76 [95% CI, 0.72–0.80]) and 12-hour horizons (AUROC, 0.93 [95% CI, 0.92–0.94] and AUPRC, 0.68 [95% CI, 0.64–0.72]). To alert before 80% of events, P-WIN generated 0.20 alerts per patient-day at a median 10.17 hours before CDE. Compared with the existing rule-based PICU Warning Tool (alerting before 38% of events), P-WIN generated one-third the alert burden at equivalent sensitivity (0.03 vs. 0.10 alerts per patient-day).

**CONCLUSIONS:**

P-WIN accurately predicted PICU CDEs up to 12 hours in advance with low alert burden, providing a viable opportunity for shifting care from reactive rescue to proactive, resource-intensive preparation and prevention.

**KEY POINTS**

**Question**: Can a machine learning model accurately predict critical deterioration events in the PICU with sufficient lead time and acceptable alert burden for clinical implementation?

**Findings**: In this retrospective cohort study with temporal validation (*n* = 17,915 patients), an extreme gradient-boosted ensemble model (PICU Warning INdex [P-WIN]) achieved excellent discrimination (area under the receiver operating characteristic curve, 0.93–0.95) for predicting critical deterioration events 2–12 hours in advance. At equivalent sensitivity, P-WIN generated one-third the alert burden of an existing rule-based warning system (0.03 vs. 0.10 alerts per patient-day), an operationally significant improvement.

**Meaning**: Machine learning-based prediction can provide timely, accurate warnings with low alert burden, enabling a shift from reactive rescue to proactive preparation in pediatric critical care.

Cardiopulmonary deterioration is a critical, potentially modifiable event on the path to mortality in the PICU (1). Early recognition and proactive intervention are needed to improve outcomes, yet current predictive systems fall short (2–4). Previous PICU early warning systems relied on automated scores that lacked time-bound prediction horizons (5), potentially contributing to alarm fatigue. The rapid emergence of machine learning (ML) models represents significant progress, yet a recent scoping review suggests that most models are not assessed with operational metrics necessary for bedside adoption (6). Consequently, they may fail to provide time-bound, actionable signals necessary for preemptive clinical intervention and shared situation awareness.

Clinically useful prediction for rare events hinges on alert burden—the frequency of alerts relative to actionable signal (7). Unfortunately, current performance measures do not capture this real-world tradeoff. For example, the commonly reported number needed to alert (NNA) is calculated at the patient level, underestimating the true false alert burden over time during each patient’s admission. Given the large investments and limited success of moving from validated models to actionable predictions at the bedside, providing relevant alert burden estimates explicitly during model development and validation is critical.

To address the need for improved early detection systems in the PICU, we aimed to: 1) determine whether an ensemble ML model utilizing routinely captured electronic health record (EHR) data accurately predicts critical deterioration events (CDEs) in the PICU population and 2) evaluate clinical utility relative to existing tools by estimating expected alert burden through a novel retrospective analysis designed to emulate prospective deployment. We hypothesized that an ML PICU deterioration model could achieve higher discrimination and lower alert burden than existing PICU early warning systems.

## METHODS

### Overview

We applied ML techniques to develop and validate a multivariable prediction model for PICU clinical deterioration and subsequently performed a prospective alert analysis in a temporally distinct cohort. The Institutional Review Board at the Children’s Hospital of Philadelphia (CHOP) reviewed and determined this study to be exempt research. This study follows the Transparent Reporting of a Multivariable Prediction Model for Individual Prognosis or Diagnosis plus Artificial Intelligence reporting guidelines (8).

### Setting and Population

This retrospective study involved patients admitted to the CHOP Philadelphia Campus PICU from 2014 to 2022. CHOP Philadelphia Campus is a freestanding quaternary children’s hospital with approximately 100 licensed PICU beds. Existing situation awareness processes and tools included twice per day unit wide huddles and a noninterruptive automated, rule-based PICU Warning Tool (5, 9) that identified patients with high-risk patient factors that precede clinical deterioration. A separate cardiac ICU that cares for post-surgical and medical cardiac patients and a separate neonatal ICU were not included because of different practice models and deterioration response systems.

We included patients 0–24 years old admitted to the CHOP PICU between January 1, 2014, and December 31, 2022. Patients 22–24 years old were included to capture greater than 99% of PICU admissions, ensuring a sample representative of actual care. We excluded patients with PICU stay durations under 24 hours. We combined admissions into one continuous admission if they occurred during the same hospital encounter, and the subject left the PICU for less than 24 hours.

We divided the population into two cohorts: 1) a development dataset consisting of all PICU admissions between 2014 and 2020 and 2) a temporal external validation dataset consisting of PICU admissions between 2021 and 2022, which was used for model evaluation and alert burden analysis. The external validation set excluded samples from hospitalizations that started in the development set period.

### Critical Deterioration Events

The primary outcome was the occurrence of a CDE. A CDE was the occurrence of either: 1) cardiopulmonary resuscitation (CPR), 2) initiation of extracorporeal membrane oxygenation (ECMO), 3) “dilute” (10 µg/mL concentration) epinephrine bolus administration, or 4) unplanned endotracheal intubation (UEI). This composite outcome was intended to capture acute escalations of support that signal significant shifts from existing care and require urgent, coordinated team action. These events are ideal targets for a system designed to improve shared situation awareness and enable just-in-time preparation (10–15). We defined UEI as any intubation that persisted for greater than or equal to 48 hours to exclude elective/procedural airway management.

PICU admissions were categorized into CDE admissions (those with at least one CDE during the admission) and non-CDE admissions. For model training, we excluded CDE admissions with only a CDE that occurred within the first 16 hours of admission to improve baseline data availability. In CDE admissions, subsequent CDEs representing new deteriorations (e.g., cardiac arrest in an already intubated patient) were included to capture deterioration despite current therapy. CDE events that occurred within 8 hours of a prior CDE event were excluded because they were considered part of the same deterioration trajectory. For example, a patient who experienced cardiac arrest and was cannulated onto ECMO during CPR would only have the CPR CDE included for model training. If CDE admissions involved multiple CDE that met the above criteria, they were included as separate samples for training. For non-CDE samples, a single nonevent time point per non-CDE admission (admissions devoid of any CDE) was selected randomly as the prediction target, excluding the first 16 hours after admission and the final 6 hours before discharge. This admission-level stratification and control down-sampling strategy was prioritized during training to reduce data leakage and mitigate class imbalance, reserving the comprehensive, continuous-time evaluation for the subsequent alert burden analysis.

### Data Source and Feature Development

CDE data, including the type of event and corresponding datetime, were obtained from a local cardiac arrest database (CPR), local Virtual Pediatric Systems registry (endotracheal intubation, ECMO cannulation time), and medication administration records (16) . All other clinical data were extracted from the local EHR (Epic Systems, Verona, WI). All features were generated from structured data elements related to patient demographics, medication administrations, laboratory results, and vital signs captured in flowsheet data. Data processing methods are described in **Supplementary Methods** (https://links.lww.com/CCX/B615). The training pipeline included 650 medication features, 329 laboratory result features, 149 flowsheet features, and two demographic features (age and sex).

### Ensemble Machine Learning Model Development

We developed an ensemble of extreme gradient boosting (XGB) decision tree models (17) trained to predict the probability of a CDE at 12, 8, 6, 4, 2, and 1 hour before the target event. We used ten-fold stratified cross-validation for training and hyperparameter optimization. The final ensemble model averages predictions of these six horizon predictions and is referred to as the PICU Warning INdex (P-WIN) model.

Ensemble models were also developed using Random Forest (18) and L1-Regularized (Least Absolute Shrinkage and Selection Operator [LASSO]) Logistic Regression (19) models. Finally, to assess the effectiveness of a small number of features, we trained ensembles of XGB models that incorporated only the top ten, 20, or 30 features, based on mutual information with the outcome, for each horizon model. The resulting ensemble models included 12, 24, and 42 unique features, respectively.

### Model Evaluation

Model performance, including the area under the receiver operating characteristic curve (AUROC), the area under the precision-recall curve (AUPRC), specificity, sensitivity, and positive predictive value, was assessed in the validation cohort. The 95% CIs were calculated using 2000 stratified bootstrap replicates. We performed a model fairness analysis by assessing the P-WIN model for variance across demographic factors including sex, race, ethnicity, insurance category, and age category. We conducted sensitivity analyses evaluating model performance in the validation cohort: 1) excluding admissions involving patients that also had admissions in the development cohort and 2) excluding admissions that involved patients older than 21 years. For all model comparisons, we tested for statistically significant differences in model AUROC performance using DeLong method (20). All statistical comparisons were performed at a significance level of *p* value of less than 0.05 corrected for multiple comparisons using false discovery rate control (Benjamini-Hochberg method) (21).

We assessed which features contributed most heavily to the model’s predictions on the external validation set using SHapley Additive exPlanations (22) values, which measure the magnitude and directionality of each feature’s influence on the model’s risk prediction.

### Alert Burden Analysis

In prospective implementation, predictive models can generate a predicted probability of the outcome at varied frequencies and, if the probability exceeds a defined threshold, generate an alert that may trigger an action or workflow change.

We emulated this process in the external validation cohort. We computed P-WIN predicted probabilities for CDE at three specified frequencies (every 1, 2, or 12 hr) for the entire duration of each PICU admission. For each prediction, an alert was generated if the predicted probability exceeded a specified alert threshold. Following an alert, we implemented a refractory period (0, 12, or 24 hr) during which no subsequent alerts could be generated for that patient. We also instituted a 24-hour censoring period following each CDE. The refractory and censoring period mimic potential situation awareness workflows, in which repeated alerts in rapid succession or immediately after a target event may not be desired.

We compared P-WIN’s performance and alert burden to the existing PICU Warning Tool alerts that generated in the EHR. Throughout the admissions in our external validation dataset, we generated an hourly binary indicator for whether the patient had a PICU Warning Tool alert triggered in the past hour. Alerts were subjected to the same refractory period and post-CDE censoring as P-WIN alerts.

For each combination of a prediction interval, refractory period, and alert threshold, we calculated the average number of alerts per patient-day across admissions. We also calculated the percentage of CDE that had an alert within 24 hours before the event. CDE occurring in the first 16 hours of admission were included (in contrast to model development and validation).

By varying the alert threshold across a range of values, we estimated the tradeoff between alert burden and the percent of CDE that had an alert before the event. To compare this approach with prior literature, we also calculated the NNA at the admission level, replicating previously published approaches (5, 23).

## RESULTS

**Figure 1.**
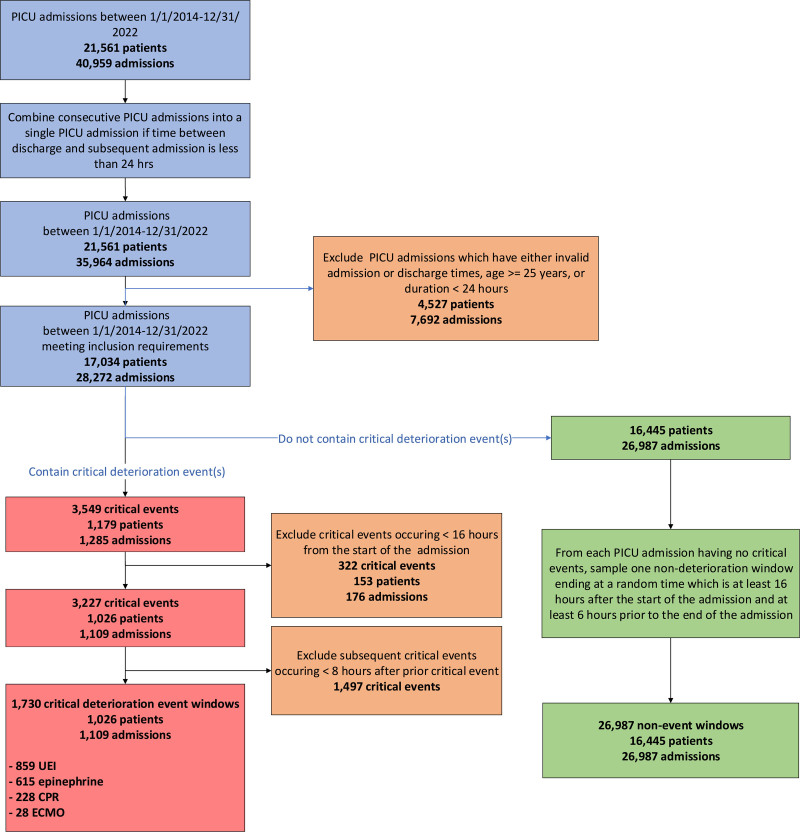
Flowchart of the cohort inclusion and exclusion process. CPR = cardiopulmonary resuscitation, ECMO = extracorporeal membrane oxygenation, UEI = unplanned endotracheal intubation.

The development cohort (2014–2020) included 12,771 patients over 21,141 admissions, of whom 751 (6%) experienced at least one CDE (**Fig. 1**). The total 1245 CDE included 177 CPR (14%), 17 ECMO (1%), 413 epinephrine boluses (33%), and 638 UEI (51%) events. The external validation cohort (2021–2022) included 5144 patients over 6929 admissions, of which 285 (6%) experienced at least one CDE. The total 484 CDE included 51 CPR (11%), 11 ECMO (2%) events, 201 epinephrine boluses (42%), and 221 UEI (46%) events. Patient demographics for the development and validation cohort are summarized in **Table 1**. Comparison of demographic details between admissions with and without CDE are included in **Supplementary Table 1** (https://links.lww.com/CCX/B615).

**TABLE 1.** Demographic Characteristics of the Development and Temporal External Validation Cohorts

Variable	Development Cohort	Validation Cohort
Number of patients	12,771	5,144
Sex = female, *n* (%)		
Yes	7,090 (56)	2,851 (56)
No	5,681 (45)	2,293 (45)
Race, *n* (%)		
White	6,231 (49)	2,426 (47)
Black	3,549 (28)	1,498 (29)
Other^a^	2,705 (21)	1,085 (21)
Unknown^b^	90 (1)	53 (1)
Conflict^c^	196 (2)	82 (2)
Hispanic or Latino ethnicity, *n* (%)		
Yes	1,703 (13)	807 (16)
No	10,939 (86)	4,273 (83)
Unknown^b^	91 (1)	55 (1)
Conflict^c^	38 (< 1)	9 (< 1)
Age category, *n* (%)		
Neonate (< 28 d)	78 (1)	35 (1)
Infant (> 27 d to < 2 yr)	4,925 (39)	1,652 (32)
Child (2–11 yr)	4,712 (37)	2,053 (40)
Adolescent (12–18 yr)	2,688 (21)	1,213 (24)
Adult (19–24 yr)	368 (3)	191 (4)
Insurance category, *n* (%)		
Private	6,396 (50)	2,539 (49)
Medicaid	5,223 (41)	2,378 (46)
Medicare	43 (< 1)	12 (< 1)
Charity	19 (< 1)	15 (< 1)
Selfpay/other	334 (3)	117 (2)
Unknown^b^	756 (6)	83 (2)
CDE outcomes, *n* (%)		
Experienced no CDE	12,020 (94)	4,887 (95)
Experienced at least one CDE	751 (6)	285 (6)
Experienced unplanned endotracheal intubation	493 (4)	164 (3)
Experienced epinephrine	275 (2)	127 (3)
Experienced cardiopulmonary resuscitation	110 (1)	41 (1)
Experienced extracorporeal membrane oxygenation	16 (< 1)	10 (< 1)

CDE = critical deterioration event.

a The “other” race category contains patients whose self-reported race in the electronic health record (EHR) was “Asian,” “Indian,” “Native Hawaiian or other Pacific Islander,” “American Indian or Alaska Native,” or “other.”

b The “unknown” race and ethnicity categories comprise patients whose self-reported race in the EHR was “choose not to disclose,” “unknown,” “asked but unknown,” or “refused.”

c The “conflict” category race and ethnicity consists of patients who had several different race or ethnicity records in the EHR during their first hospitalization.

Table presenting the demographic characteristics and outcome counts in the development (2014–2020) and temporal external validation (2021–2022) cohorts. Demographic information, such as age and insurance status, is based on the values at the time of hospital admission for the encounter that included the relevant PICU admissions included in the development or validation cohorts.

A composite of either: 1) cardiopulmonary resuscitation, 2) initiation of extracorporeal membrane oxygenation, 3) administration of a bolus dose of “dilute” (10 µg/mL concentration) epinephrine, or 4) unplanned endotracheal intubation.

### P-WIN Model Performance

**Table 2** reports AUROC and AUPRC in the external validation dataset at 2- and 12-hour time horizons before the prediction target for all ensemble models. P-WIN outperformed the random forest, LASSO, and parsimonious ensemble models (2-hr P-WIN: AUROC, 0.95 [95% CI, 0.94–0.96] vs. 2-hr LASSO: AUROC, 0.93 [95% CI, 0.91–0.94]; *p* < 0.01 vs. 2-hr RF: AUROC, 0.93 [95% CI, 0.91–0.94]; *p* < 0.01). It was also superior at all other time horizons (**Supplementary Tables 2** and **3**, https://links.lww.com/CCX/B615).

**TABLE 2.** Predictive Performance of the Ensemble Models on the Temporal External Validation Dataset, at 12-Hour and 2-Hour Prediction Horizons

Model	12-hr Horizon		2-hr Horizon	
	AUROC	AUPRC	AUROC	AUPRC
Extreme gradient boosting ensemble (PICU Warning INdex)	0.93 (0.92–0.94)^b^	0.68 (0.64–0.72)	0.95 (0.94–0.96)^b^	0.76 (0.72–0.80)
Random forest ensemble	0.90 (0.88–0.92)^a^	0.61 (0.56–0.65)	0.92 (0.91–0.94)^a^	0.69 (0.65–0.74)
Least Absolute Shrinkage and Selection Operator ensemble	0.90 (0.89–0.92)^a^	0.59 (0.54–0.63)	0.93 (0.91–0.94)^a^	0.66 (0.62–0.71)
Parsimonious ensemble models				
12-feature	0.87 (0.85–0.89)^a^	0.52 (0.47–0.56)	0.90 (0.89–0.92)^a^	0.61 (0.56–0.65)
24-feature	0.87 (0.85–0.89)^a^	0.55 (0.51–0.60)	0.91 (0.90–0.93)^a^	0.64 (0.60–0.69)
42-feature	0.88 (0.86–0.90)^a^	0.57 (0.52–0.61)	0.92 (0.90–0.94)^a^	0.66 (0.62–0.71)

AUPRC = area under the precision-recall curve, AUROC = area under the receiver operating characteristic curve.

a Statistically significant difference (DeLong test *p* < 0.05) compared with the PICU Warning INdex (P-WIN) extreme gradient boosting (XGB) ensemble model at the same prediction horizon.

b Indicates the best performance across models within a fixed prediction horizon.

A performance comparison of all ensemble models in the validation dataset at a 12-hr and 2-hr horizon. The prevalence of events in the external validation dataset was 7%.

XGB ensemble model (the primary model also referred to as P-WIN throughout the article). Parsimonious ensemble models are XGB ensemble models with fewer features than the P-WIN model (550 features).

**Figure 2.**
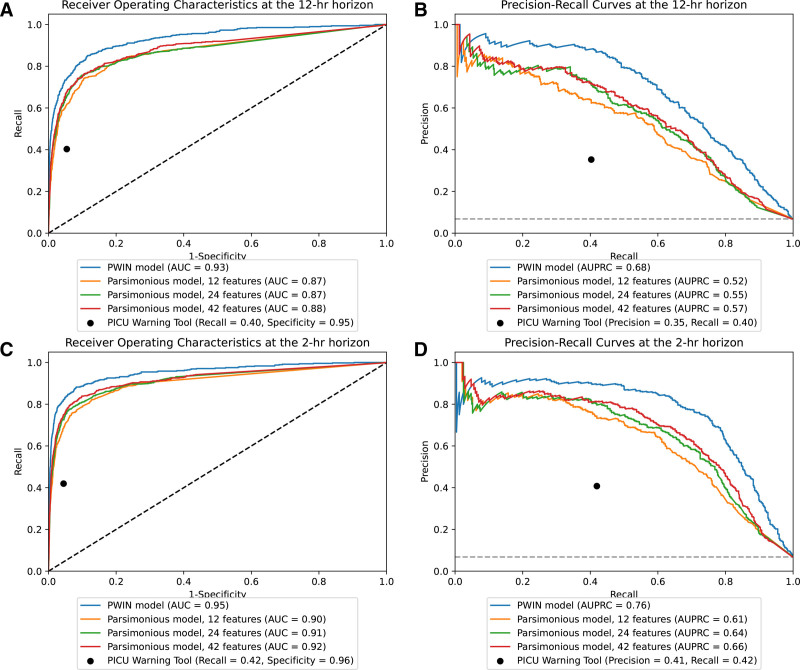
Predictive performance of the PICU Warning INdex (P-WIN) and parsimonious models in the external validation cohort. Receiver operating characteristics and precision-recall curves for the P-WIN extreme gradient boosting ensemble model (primary ensemble model incorporating 550 features) and three parsimonious models at 12-hr (**A** and **B**) and 2-hr (**C** and **D**) prediction horizons on the external validation dataset. The prevalence of events in the external validation dataset was 7%. In *each plot*, the *black dot* indicates the performance of the binary PICU Warning Tool predictions. AUC = area under the curve, AUPRC = area under the precision-recall curve.

P-WIN AUPRC was 11 times (2-hr) and nine times (12-hr) higher than CDE prevalence (7%), and P-WIN outperformed the binary PICU Warning Tool predictions at all time horizons (**Fig. 2**). At 80% sensitivity, P-WIN had an NNA of 1.6 (2-hr) and 2.4 (12-hr). **Supplementary Table 4** (https://links.lww.com/CCX/B615) reports P-WIN performance at additional sensitivity and specificity thresholds. P-WIN achieved an AUROC above 0.90 for all horizons for each individual event type in the validation cohort (**Supplementary Table 5**, https://links.lww.com/CCX/B615).

P-WIN had similar performance after excluding patients in the validation dataset that were seen in the development dataset and excluding patients in the validation dataset involving patients older than 21 years (**Supplementary Table 6**, https://links.lww.com/CCX/B615). In model fairness analysis, P-WIN had statistically significant greater AUROC for children younger than 28 days than children 2–11 years (reference group) at all prediction horizons (**Supplementary Table 7**, https://links.lww.com/CCX/B615).

P-WIN uses 550 EHR-derived variables (335 variables from administered medications, 110 variables from laboratory results, 103 variables from flowsheet vital signs, and two demographic variables; **Supplementary Table 8**, https://links.lww.com/CCX/B615). The most important features across horizon models clustered into three EHR based physiologic signatures: respiratory physiology and support, neurologic status, and hemodynamic stability. **Supplementary Figures 1** and **2** (https://links.lww.com/CCX/B615) illustrate variable importance for P-WIN 2- and 12-hour horizon models. **Supplementary Table 9** (https://links.lww.com/CCX/B615) provides a list of all variables included across the three parsimonious models.

### Alert Burden Analysis Results Using External Validation Data

**Figure 3.**
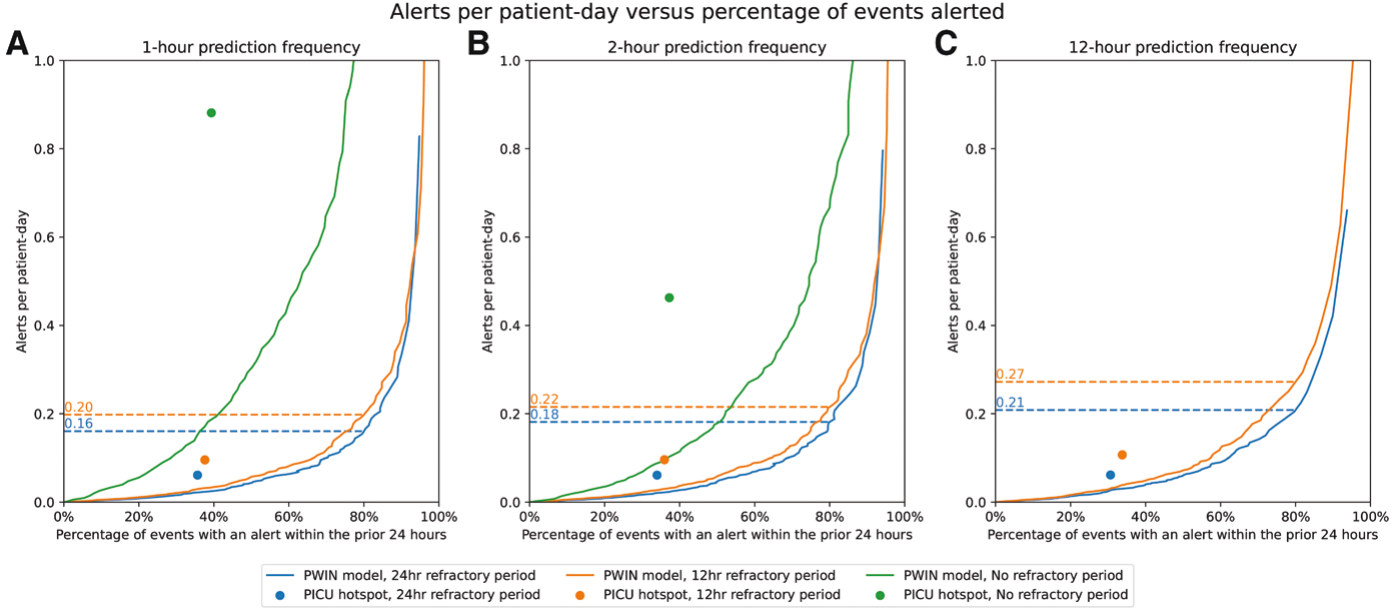
The average alerts per patient-day vs. the percentage of critical deterioration events (CDEs) for which an alert was raised in the preceding 24 hr, at all alert thresholds, using various prediction frequencies and refractory period lengths. Predictions were generated every 1 hr (**A**), 2 hr (**B**), or 12 hr (**C**) and using either no refractory period or 12- or 24-hr refractory periods. A refractory period is a duration of time after an alert during which additional predictions that may meet the alert threshold do not trigger an alert. Note that only two *curves* appear on the third *plot*, as a 12-hr refractory period is equivalent to no refractory period for 12-hr prediction frequency. *Dashed lines* indicate threshold values for which 80% of CDEs had alerts raised. *Dots* indicate the performance of the binary PICU Warning Tool predictions. P-WIN = PICU Warning INdex.

The external validation cohort of 6,929 admissions involved 5,144 patients and 60,251 patient-days. In 301 admissions (4%), 285 patients experienced 504 CDEs. **Figure 3** illustrates the estimated average alerts per patient-day vs. the percentage of CDE with an alert in the preceding 24 hours across a range of prediction thresholds and prediction frequencies to demonstrate the tradeoff of detection and alert burden.

For example, with hourly predictions and 12-hour refractory period, P-WIN generated ~10 alerts per 12-hour shift to capture 80% of CDE in a 100-bed unit. This alert burden corresponded to an NNA of 9.29, at the admission level. At this threshold, alerts before CDE occurred at a median 10.17 hours (interquartile range, 5.20–17.30 hr) before the CDE. **Supplementary Table 10** (https://links.lww.com/CCX/B615) provides alerts per patient-day estimates at alert thresholds that lead to alerts before 80%, 90%, and 95% of CDE. **Supplementary Figure 3** (https://links.lww.com/CCX/B615) shows alerts per patient-day plots for parsimonious models at similar varied thresholds and refractory periods.

We benchmarked P-WIN directly against the existing PICU Warning Tool (**Supplementary Table 11**, https://links.lww.com/CCX/B615). P-WIN demonstrated superior performance in two scenarios. When matching the alert burden of the existing tool (0.10 alerts/patient-day), P-WIN detected nearly twice as many events (66% [95% CI, 62–70%] vs. 38% [95% CI, 33–42%]). When fixed to the same percentage of detected events as the existing tool, P-WIN generated lower alert burden (0.03 alerts per patient-day [95% CI, 0.03–0.04 alerts per patient-day] vs. 0.10 alerts per patient-day [95% CI, 0.09–0.10 alerts per patient-day]). These alert burdens corresponded to significantly lower NNA at the admission level (P-WIN NNA (6.0 [95% CI, 5.2–6.9] vs. 9.4 [95% CI, 8.3–10.6]).

## DISCUSSION

We developed and validated P-WIN, an ensemble ML model that predicts CDEs in the PICU using routinely collected EHR data. P-WIN demonstrated high discrimination and precision across 1–12-hour time horizons in a temporally distinct external validation cohort. In a comparative alert analysis, P-WIN significantly outperformed existing automated warning systems, detecting more CDE while generating fewer alerts. Collectively, these findings suggest that P-WIN provides the timely, actionable prediction required for high-resource interventions like interdisciplinary huddles and just-in-time simulation.

P-WIN addresses limitations of existing predictive approaches. While generalized mortality prediction or risk stratification systems are useful for benchmarking, they are decoupled from the actionable rescue events that require immediate preparation (24–26). Similarly, models predicting single events, such as cardiac arrest, leave users to reconcile disparate, single-event risk scores across outcomes, limiting utility for general situation awareness (27). Composite rule-based, high-risk identification systems have improved outcomes when paired with frequent situation awareness huddles (2, 3, 5). However, inherently low sensitivity and high false-alert rates limit their use for triggering more time-intensive interventions. Finally, generalized multicenter algorithms such as the pediatric Critical Event Risk Evaluation and Scoring Tool demonstrate fair discrimination in the ICU setting (reported AUROC < 0.8)—possibly by missing unit specific practice patterns and temporal context that are critical for ICU deterioration (23).

P-WIN bridges these gaps by targeting a composite measure that captures a broad spectrum of relevant ICU events surrounding physiologic deterioration. The specified CDE events are acute escalations in patient support that can benefit from coordinated care through bedside huddles, actionable checklists, or even just-in-time simulation to support team performance. The ensemble of time-horizon-specific models with a broad set of EHR features enables the model to capture relevant clinical context at sufficient lead times for clinical usability (28). This comprehensive approach proved superior to the existing PICU Warning Tool, detecting nearly double the number of CDE with similar alert burden. The performance differential highlights the value of high-dimensional data to capture complex, site-specific practice patterns that simpler, “generalizable” models often miss. Our results may suggest that modeling local complexity is a prerequisite for achieving clinically meaningful precision. Thus, this study implies that an effective path to implementation may not be a single, universal algorithm, but rather a federated system that enables powerful, locally attuned models (29). While the inclusion of clinician-initiated features introduces potential bias from provider concern, the predominance of objective vital signs among top predictors suggests P-WIN remains grounded in intrinsic physiology. Reassuringly, we observed no significant performance disparities across demographic groups, although continuous evaluation for algorithmic bias will be critical during prospective deployment.

It is equally important that a predictive model for situation awareness does not exacerbate alert fatigue (30, 31). Unfortunately, estimating alert burden using the commonly reported NNA (4, 7) is inadequate for understanding true operational impact. NNA, commonly calculated at the per-patient or per-admission level, ignores the frequency of repeated false alarms during a patient’s stay (23). While the PICU Warning Tool’s NNA was only 1.6 times that of P-WIN, it generated more than three times the alerts per patient-day. In contrast to NNA, alerts per patient-day provides more realistic estimates of alert burden that accounts for multiple false alerts per patient during a stay and varied model implementation parameters. This approach enables stakeholders to assess model utility for situation awareness across diverse workflows and demonstrates a more rigorous standard for evaluating implementation readiness at the model development stage.

Finally, the model’s sustained accuracy across time horizons optimizes for workflow integration. This extended lead-time supports existing operational routines such as once-per-shift safety huddles rather than attempting to identify actively deteriorating patients that require immediate reactive response. P-WIN’s high accuracy and timely prediction can enable proactive structured bedside interdisciplinary huddles, facilitating subsequent actions such as task list generation, cognitive walkthroughs, and even just-in-time simulation. By providing a reliable planning window, P-WIN transforms a warning signal from a source of alarm fatigue to a catalyst for coordinated, proactive team preparation.

This study has several limitations. First, the single-center design inherently limits the generalizability of our findings. Therefore, external validation across diverse PICU settings, including prospective silent evaluation, is a critical next step. Second, our validation, although performed on a temporally distinct cohort, was retrospective. A retrospective alert simulation cannot fully replicate the complex feedback loops of a live clinical deployment, where alerts may influence clinician behavior and subsequent documentation. Finally, our model was not trained in certain specialized populations, such as cardiac ICU patients, and its performance in these groups is unknown.

## CONCLUSIONS

An ML deterioration prediction model, P-WIN, delivers accurate, timely predictions with a low alert burden, providing a reliable foundation for high-resource situation awareness interventions. Future work will include a silent implementation assessment to validate model performance and evaluate validity of alert analysis. Subsequent multicenter study will assess the impact of model implementation on patient-centered outcomes.

## ACKNOWLEDGMENTS

We thank the staff of the Children’s Hospital of Philadelphia Critical Care Center for Evidence and Outcomes for their efforts in abstracting and coding the local Virtual Pediatric Systems data used to prepare this report.

## Supplementary Material
