# Supplementary material for: Machine Learning-Based Prediction of Critical Deterioration in the PICU
Source: Crit Care Explor. 2026 Apr 22;8(4):e1397. doi: 10.1097/CCE.0000000000001397 (PMC13098776; doi:10.1097/CCE.0000000000001397)
Supplement: Supplementary file 1 [file cc9-8-e1397-s001.pdf]

Supplementary materials

Table of Contents

Supplementary materials 1

Supplementary Methods 2

Data processing and feature extraction methods 2

Feature sampling and forward-filling 2

Supplementary Tables 3

Supplementary Table 1. Demographic characteristics of patients and samples with and without a Critical Deterioration Event. 3

Supplementary Table 2. Performance of the Ensemble Models on the External Validation Dataset at varied horizons. 4

Supplementary Table 3. Comparison of P-WIN ensemble model and parsimonious ensemble models. 5

Supplementary Table 4. Sensitivity, Specificity, and PPV scores for the P-WIN XGB Ensemble Model on the external validation set. 6

Supplementary Table 5. Performance of the P-WIN XGB ensemble model on the external validation dataset, by individual event type. 8

Supplementary Table 6. Sensitivity analyses results for the PWIN model on the external validation dataset. 9

Supplementary Table 7. P-WIN Model Fairness Evaluation Results. 10

Supplementary Table 8. Counts of EHR variables used by each ensemble model. 11

Supplementary Table 9. Model variables used by any of the 12-, 24-, or 42-variable parsimonious models. 12

Supplementary Table 10. Retrospective Alert Burden Study Results..............................13

Supplementary Figures 15

Supplementary Figure 1. SHapley Additive exPlanations (SHAP) value results for the predictions of the XGBoost P-WIN 12-hour horizon model on the external validation dataset. 15

Supplementary Figure 2. SHapley Additive exPlanations (SHAP) value results for the predictions of the XGBoost P-WIN 2-hour horizon model on the external validation dataset. 16

Supplementary Figure 3. The average alerts per patient-day versus the percentage of CDEs for which an alert was raised, for various parsimonious models at all alert thresholds, using various prediction frequencies and refractory period lengths. 17

Tripod+AI Checklist 19

References 21

Supplementary Methods

Data processing and feature extraction methods

Medications were mapped from National Drug Codes (NDC) to RxNorm[1] active ingredients and Veterans Administration (VA) drug classes[2] using the RxNav tool hosted by the National Library of Medicine at the National Institutes of Health (https://lhncbc.nlm.nih.gov/RxNav/). Laboratory tests were mapped to standardized Logical Observation Identifiers Names and Codes (LOINC)[3]. LOINC codes for which fewer than 95% of results were numerical were excluded. Vital signs were mapped to features based on the flowsheet display name. For all data sources, features which appeared for fewer than 0.5% of event window samples were dropped. After applying this filter, we were left with 458 VA class features, 371 active ingredient features, 329 lab result features, and 149 vital sign features.

Feature sampling and forward-filling

Each horizon's component model was trained on a collection of model variables obtained by aggregating the EHR features over a window ending at its respective prediction horizon time. For each medication feature (active ingredient or VA class), we derived a binary variable indicating whether any medication corresponding to that feature was administered in the 4 hours prior to the prediction horizon. For each lab result or numerical vital sign, we derived a numerical feature consisting of the median value over the 30-minute window prior to the prediction horizon, where null values were forward-filled for 4 hours. Variables for categorical vital signs consisted of the most recent value, with null values again forward-filled for 4 hours.

Supplementary Tables

Supplementary Table 1. *Demographic characteristics of patients and samples with and without a Critical Deterioration Event.*

	Non-Critical Deterioration Admissions	Critical Deterioration Admissions	Odds Ratio (95% Confidence Interval)
**Samples n (%)**	26,987 (100)	1,730 (100)	
**Sex = Female, n (%)**			
Yes	11,982 (44)	803 (46)	1.09 (0.98 to 1.20)
No	15,005 (56)	927 (54)	Reference
**Race, n (%)**			
White	12,839 (48)	742 (43)	Reference
Black	7,576 (28)	449 (26)	1.02 (0.91 to 1.15)
Other[a]	5,970 (22)	505 (29)	1.46 (1.30 to 1.65)
Unknown[b]	193 (1)	14 (1)	1.25 (0.69 to 2.08)
Conflict[c]	409 (2)	20 (1)	0.84 (0.52 to 1.30)
**Hispanic or Latino ethnicity, n (%)**			
Yes	4,319 (16)	293 (17)	1.07 (0.94 to 1.21)
No	22,409 (83)	1,426 (82)	Reference
Unknown[b]	187 (1)	6 (<1)	0.50 (0.20 to 1.03)
Conflict[c]	72 (<1)	5 (<1)	1.08 (0.38 to 2.43)
**Age category, n (%)**			
Neonate (< 28 days)	99 (<1)	30 (2)	5.33 (3.47 to 7.97)
Infant (>27 days, <2yrs)	8,471 (31)	646 (37)	1.39 (1.24 to 1.55)
Child (2yrs - 11yrs)	11,547 (43)	635 (37)	Reference
Adolescent (12yrs - 18yrs)	5,703 (21)	418 (24)	1.33 (1.17 to 1.51)
Adult (19yrs – 24yrs)	1,167 (4)	1 (<1)	0.02 (0.00-0.07)
**Insurance Category, n (%)**			
Private	13,077 (49)	723 (42)	Reference
Medicaid	12,196 (45)	789 (46)	1.17 (1.05 to 1.3)
Medicare	123 (1)	9 (1)	1.33 (0.63 to 2.48)
Charity	44 (<1)	1 (<1)	0.41 (0.02 to 1.9)
Selfpay/Other	686 (3)	121 (7)	3.19 (2.58 to 3.91)
Unknown	861 (3)	87 (5)	1.81 (1.43 to 2.28)

Table presenting the demographic data of PICU admission samples categorized by presence of a critical deterioration event during the PICU admission. Demographic information such as age and insurance status are based on the values at the time of hospital admission for the encounter that included the relevant PICU admission.

[a]*The "Other" Race category contains patients whose self-reported race in the EHR was "Asian", "Indian", "Native Hawaiian or Other Pacific Islander", "American Indian or Alaska Native", or "Other".*

[b]*The "Unknown" Race and Ethnicity categories comprise patients whose self-reported race in the EHR was "Choose note to disclose", "Unknown", "Asked but unknown", or "Refused".*

[c]*The "Conflict" category Race and Ethnicity consist of patients who had several conflicting records in the EHR during their first hospitalization.*

Supplementary Table 2. *Performance of the Ensemble Models on the External Validation Dataset at varied horizons.*

Prediction horizon	XGB (P-WIN)	RF	LASSO
12 hour			
AUROC (95% CI)	0.93 (0.92-0.94) [b]	0.90 (0.88-0.92)[a]	0.90 (0.89-0.92) [a]
AUPRC (95% CI)	0.68 (0.64-0.72)	0.61 (0.56-0.65)	0.59 (0.54-0.63)
8 hour			
AUROC (95% CI)	0.94 (0.92-0.95) [b]	0.91 (0.90-0.93) [a]	0.91 (0.89-0.92) [a]
AUPRC (95% CI)	0.71 (0.67-0.75)	0.64 (0.60-0.68)	0.61 (0.57-0.66)
6 hour			
AUROC (95% CI)	0.94 (0.93-0.96) [b]	0.92 (0.91-0.94) [a]	0.92 (0.90-0.93) [a]
AUPRC (95% CI)	0.73 (0.69-0.77)	0.67 (0.63-0.71)	0.63 (0.58-0.67)
4 hour			
AUROC (95% CI)	0.95 (0.94-0.96) [b]	0.92 (0.91-0.94) [a]	0.92 (0.90-0.93) [a]
AUPRC (95% CI)	0.73 (0.69-0.77)	0.67 (0.62-0.71)	0.64 (0.59-0.68)
2 hour			
AUROC (95% CI)	0.95 (0.94-0.96) [b]	0.92 (0.91-0.94) [a]	0.93 (0.91-0.94) [a]
AUPRC (95% CI)	0.76 (0.72-0.80)	0.69 (0.65-0.74)	0.66 (0.62-0.71)
1 hour			
AUROC (95% CI)	0.96 (0.95-0.97) [b]	0.94 (0.92-0.95) [a]	0.93 (0.92-0.94) [a]
AUPRC (95% CI)	0.78 (0.75-0.82)	0.72 (0.68-0.76)	0.68 (0.64-0.73)

Predictive performance of the XGBoost (P-WIN), random forest, and LASSO ensemble models on the external validation dataset at various prediction horizons. The prevalence of events in the external validation dataset was 6.81%.

[a]*Statistically significant difference (Delong test P<0.01) compared with the XGB model at the same prediction horizon.*

[b]*Indicates the best performance across models within a fixed prediction horizon.*

Supplementary Table 3. *Comparison of P-WIN ensemble model and parsimonious ensemble models.*

Prediction horizon	P-WIN ensemble model	12 features ensemble model	24 features ensemble model	42 features ensemble model
12 hour				
AUROC (95% CI)	0.93 (0.92-0.94)	0.87 (0.85-0.89) [a]	0.87 (0.85-0.89) [a]	0.88 (0.86-0.90) [a]
AUPRC (95% CI)	0.68 (0.64-0.72)	0.52 (0.47-0.56)	0.55 (0.51-0.60)	0.57 (0.52-0.61)
8 hour				
AUROC (95% CI)	0.94 (0.92-0.95)	0.89 (0.87-0.90) [a]	0.89 (0.87-0.91) [a]	0.91 (0.89-0.92) [a]
AUPRC (95% CI)	0.71 (0.67-0.75)	0.57 (0.52-0.62)	0.59 (0.55-0.64)	0.62 (0.58-0.67)
6 hour				
AUROC (95% CI)	0.94 (0.93-0.96)	0.90 (0.88-0.91) [a]	0.90 (0.88-0.92) [a]	0.92 (0.90-0.93) [a]
AUPRC (95% CI)	0.73 (0.69-0.77)	0.59 (0.55-0.64)	0.62 (0.57-0.67)	0.64 (0.59-0.68)
4 hour				
AUROC (95% CI)	0.95 (0.94-0.96)	0.90 (0.88-0.92) [a]	0.90 (0.89-0.92) [a]	0.92 (0.90-0.93) [a]
AUPRC (95% CI)	0.73 (0.69-0.77)	0.59 (0.54-0.63)	0.61 (0.56-0.66)	0.63 (0.59-0.68)
2 hour				
AUROC (95% CI)	0.95 (0.94-0.96)	0.90 (0.89-0.92) [a]	0.91 (0.90-0.93) [a]	0.92 (0.90-0.94) [a]
AUPRC (95% CI)	0.76 (0.72-0.80)	0.61 (0.56-0.65)	0.64 (0.60-0.69)	0.66 (0.62-0.71)
1 hour				
AUROC (95% CI)	0.96 (0.95-0.97)	0.91 (0.90-0.93)[a]	0.93 (0.91-0.94) [a]	0.93 (0.92-0.95) [a]
AUPRC (95% CI)	0.78 (0.75-0.82)	0.64 (0.59-0.68)	0.67 (0.63-0.71)	0.69 (0.65-0.73)

Comparison of the external validation dataset performance of the full de novo P-WIN XGB ensemble model and three P-WIN XGB parsimonious models, for various prediction horizons. The prevalence of critical deterioration events in the development dataset was 6.81%.
The parsimonious models are each ensembles of six horizon-specific models, and rely on 12, 24, and 42 features, respectively.
[a]Statistically, significant difference (Delong test P<0.05) compared with the XGB ensemble model at the same prediction horizon.

Supplementary Table 4. *Sensitivity, Specificity, and PPV scores for the P-WIN XGB Ensemble Model on the external validation set.*

Target sensitivity of 0.95

Prediction horizon	Sensitivity	Specificity	PPV
12 hr	0.95 (0.93 to 0.97)	0.62 (0.61 to 0.63)	0.15 (0.15 to 0.16)
8 hr	0.95 (0.93 to 0.97)	0.57 (0.56 to 0.58)	0.14 (0.14 to 0.14)
6 hr	0.95 (0.93 to 0.97)	0.61 (0.60 to 0.62)	0.15 (0.15 to 0.16)
4 hr	0.95 (0.93 to 0.97)	0.62 (0.61 to 0.64)	0.16 (0.15 to 0.16)
2 hr	0.95 (0.93 to 0.97)	0.73 (0.72 to 0.74)	0.20 (0.20 to 0.21)
1 hr	0.95 (0.93 to 0.97)	0.77 (0.76 to 0.78)	0.23 (0.22 to 0.24)

Target sensitivity of 0.90

Prediction horizon	Sensitivity	Specificity	PPV
12 hr	0.90 (0.87 to 0.93)	0.78 (0.77 to 0.79)	0.23 (0.22 to 0.24)
8 hr	0.90 (0.87 to 0.93)	0.83 (0.82 to 0.84)	0.28 (0.27 to 0.29)
6 hr	0.90 (0.87 to 0.93)	0.85 (0.84 to 0.86)	0.30 (0.29 to 0.32)
4 hr	0.90 (0.87 to 0.93)	0.85 (0.84 to 0.85)	0.30 (0.29 to 0.31)
2 hr	0.90 (0.88 to 0.93)	0.87 (0.86 to 0.88)	0.34 (0.33 to 0.36)
1 hr	0.90 (0.87 to 0.93)	0.92 (0.91 to 0.92)	0.44 (0.42 to 0.46)

Target sensitivity of 0.80

Prediction horizon	Sensitivity	Specificity	PPV
12 hr	0.80 (0.76 to 0.84)	0.92 (0.91 to 0.92)	0.42 (0.40 to 0.44)
8 hr	0.80 (0.76 to 0.84)	0.94 (0.93 to 0.94)	0.49 (0.46 to 0.51)
6 hr	0.80 (0.76 to 0.84)	0.95 (0.94 to 0.96)	0.54 (0.51 to 0.57)
4 hr	0.80 (0.76 to 0.83)	0.95 (0.95 to 0.96)	0.55 (0.53 to 0.58)
2 hr	0.80 (0.77 to 0.84)	0.96 (0.96 to 0.97)	0.63 (0.60 to 0.66)
1 hr	0.80 (0.77 to 0.84)	0.98 (0.97 to 0.98)	0.70 (0.67 to 0.73)

Target specificity of 0.80

Prediction horizon	Sensitivity	Specificity	PPV
12 hr	0.90 (0.87 to 0.92)	0.80 (0.79 to 0.81)	0.25 (0.24 to 0.26)
8 hr	0.91 (0.88 to 0.94)	0.80 (0.79 to 0.81)	0.25 (0.24 to 0.26)
6 hr	0.92 (0.90 to 0.94)	0.80 (0.79 to 0.81)	0.25 (0.24 to 0.26)
4 hr	0.92 (0.89 to 0.94)	0.80 (0.79 to 0.81)	0.25 (0.24 to 0.26)
2 hr	0.93 (0.90 to 0.95)	0.80 (0.79 to 0.81)	0.25 (0.24 to 0.26)
1 hr	0.94 (0.92 to 0.96)	0.80 (0.79 to 0.81)	0.26 (0.25 to 0.27)

Target specificity of 0.90

Prediction horizon	Sensitivity	Specificity	PPV
12 hr	0.82 (0.79 to 0.86)	0.90 (0.89 to 0.91)	0.38 (0.36 to 0.40)
8 hr	0.86 (0.83 to 0.89)	0.90 (0.89 to 0.91)	0.39 (0.37 to 0.41)
6 hr	0.87 (0.84 to 0.90)	0.90 (0.89 to 0.91)	0.39 (0.37 to 0.41)
4 hr	0.87 (0.84 to 0.90)	0.90 (0.89 to 0.91)	0.39 (0.37 to 0.41)
2 hr	0.89 (0.86 to 0.92)	0.90 (0.89 to 0.91)	0.39 (0.38 to 0.41)

1 hr	0.90 (0.88 to 0.93)	0.90 (0.89 to 0.91)	0.40 (0.38 to 0.42)

Target specificity of 0.95

Prediction horizon	Sensitivity	Specificity	PPV
12 hr	0.73 (0.69 to 0.77)	0.95 (0.94 to 0.96)	0.52 (0.49 to 0.55)
8 hr	0.77 (0.73 to 0.81)	0.95 (0.94 to 0.96)	0.53 (0.50 to 0.56)
6 hr	0.80 (0.76 to 0.83)	0.95 (0.94 to 0.96)	0.54 (0.51 to 0.57)
4 hr	0.81 (0.77 to 0.85)	0.95 (0.94 to 0.96)	0.54 (0.52 to 0.57)
2 hr	0.83 (0.80 to 0.87)	0.95 (0.94 to 0.96)	0.55 (0.52 to 0.58)
1 hr	0.86 (0.83 to 0.89)	0.95 (0.94 to 0.96)	0.56 (0.53 to 0.59)

Sensitivity, specificity, and positive predictive values (PPV) scores of the PWIN ensemble XGB model on the external validation set, at various prediction horizons and using various prediction thresholds. Thresholds were chosen to achieve target sensitivity or specificity of 0.95, 0.90, and 0.80 respectively at each horizon.

Supplementary Table 5. *Performance of the P-WIN XGB ensemble model on the external validation dataset, by individual event type.*

Prediction horizon	UEI only	CPR only	Epinephrine only	ECMO only
12 hr				
AUROC (95% CI)	0.92 (0.89-0.93)	0.93 (0.89-0.97)	0.94 (0.92-0.96)	1.00 (0.99-1.00)
AUPRC (95% CI)	0.42 (0.36-0.49)	0.34 (0.24-0.49)	0.65 (0.58-0.72)	0.63 (0.40-0.89)
8 hr				
AUROC (95% CI)	0.92 (0.90-0.94)	0.91 (0.86-0.96)	0.95 (0.93-0.97)	1.00 (1.00-1.00)
AUPRC (95% CI)	0.44 (0.38-0.51)	0.31 (0.20-0.45)	0.69 (0.63-0.76)	0.67 (0.46-0.92)
6 hr				
AUROC (95% CI)	0.94 (0.92-0.95)	0.94 (0.90-0.97)	0.95 (0.93-0.97)	1.00 (1.00-1.00)
AUPRC (95% CI)	0.47 (0.41-0.54)	0.31 (0.21-0.45)	0.69 (0.63-0.76)	0.61 (0.42-0.84)
4 hr				
AUROC (95% CI)	0.94 (0.92-0.95)	0.93 (0.89-0.97)	0.96 (0.94-0.97)	1.00 (1.00-1.00)
AUPRC (95% CI)	0.48 (0.42-0.55)	0.30 (0.21-0.44)	0.69 (0.62-0.76)	0.55 (0.36-0.83)
2 hr				
AUROC (95% CI)	0.94 (0.92-0.96)	0.94 (0.89-0.97)	0.96 (0.94-0.98)	1.00 (1.00-1.00)
AUPRC (95% CI)	0.55 (0.49-0.63)	0.34 (0.24-0.49)	0.70 (0.64-0.78)	0.57 (0.39-0.85)
1 hr				
AUROC (95% CI)	0.95 (0.94-0.97)	0.94 (0.90-0.97)	0.98 (0.97-0.99)	1.00 (1.00-1.00)
AUPRC (95% CI)	0.59 (0.53-0.66)	0.32 (0.23-0.47)	0.73 (0.66-0.80)	0.62 (0.42-0.88)

Predictive performance of the P-WIN XGB ensemble model on the external validation dataset, restricted to individual event type. For each event type (UEI, CPR, epinephrine, and ECMO), only observation windows ending in an event of that type or ending in no event were included in the evaluation and other event types were excluded.

Considering only one event type at a time and excluding others: UEI events have 3.23% prevalence, CPR events have 2.94% prevalence, epinephrine events have 0.76% prevalence, and ECMO events have 0.17% prevalence.

Supplementary Table 6. *Sensitivity analyses results for the PWIN model on the external validation dataset.*

**Model**	**12-Hour Horizon**		**2-Hour Horizon**	
	**AUROC**	**AURPC**	**AUROC**	**AUPRC**
Full external validation dataset	0.93 (0.92-0.94)	0.68 (0.64-0.72)	0.95 (0.94-0.96)	0.76 (0.72-0.80)
Excluding admissions involving patients seen in development dataset	0.94 (0.92-0.95)	0.70 (0.65-0.75)	0.96 (0.94-0.97)	0.76 (0.72-0.81)
Excluding admissions involving patients aged 22-24y	0.93 (0.92-0.94)	0.68 (0.64-0.72)	0.95 (0.94-0.96)	0.76 (0.72-0.80)

Model performance results for two sensitivity analyses conducted on the external validation dataset. In the first, admissions were excluded from the external validation dataset if they involved patients having admission in the development dataset. In the second, admissions with patients aged 22-24y were excluded from the external validation dataset.

Supplementary Table 7. *P-WIN Model Fairness Evaluation Results.*

	AUROC scores for individual cohorts at various prediction horizons					
	12 hour	*8hour*	*6 hour*	*4hour*	*2 hour*	*1 hour*
Sex = Female						
Yes	0.93 (0.91 - 0.95)	0.94 (0.93 - 0.96)	0.95 (0.93 - 0.96)	0.95 (0.93 - 0.97)	0.95 (0.94 - 0.97)	0.96 (0.95 - 0.98)
No[r]	0.92 (0.90 - 0.94)	0.92 (0.90 - 0.94)	0.93 (0.92 - 0.95)	0.94 (0.92 - 0.95)	0.95 (0.93 - 0.96)	0.96 (0.94 - 0.97)
Race						
White[r]	0.92 (0.90 - 0.94)	0.93 (0.90 - 0.95)	0.94 (0.92 - 0.96)	0.94 (0.92 - 0.96)	0.95 (0.93 - 0.97)	0.97 (0.95 - 0.98)
Black	0.92 (0.89 - 0.95)	0.94 (0.92 - 0.96)	0.95 (0.92 - 0.97)	0.94 (0.91 - 0.96)	0.94 (0.91 - 0.97)	0.95 (0.92 - 0.97)
Other	0.94 (0.91 - 0.96)	0.93 (0.91 - 0.96)	0.94 (0.92 - 0.97)	0.95 (0.93 - 0.97)	0.95 (0.93 - 0.97)	0.96 (0.94 - 0.98)
Unknown	0.99 (0.95 - 1.00)**	0.98 (0.94 - 1.00)*	0.98 (0.94 - 1.00)	0.98 (0.94 - 1.00)	0.97 (0.92 - 1.00)	0.97 (0.92 - 1.00)
Hispanic or Latino						
Yes	0.94 (0.92 - 0.96)	0.94 (0.91 - 0.97)	0.95 (0.91 - 0.97)	0.95 (0.92 - 0.97)	0.96 (0.94 - 0.98)	0.97 (0.96 - 0.98)
No[r]	0.92 (0.91 - 0.94)	0.93 (0.91 - 0.94)	0.94 (0.93 - 0.95)	0.94 (0.93 - 0.96)	0.95 (0.93 - 0.96)	0.96 (0.94 - 0.97)
Age, binned						
Neonate (< 28 days)	0.99 (0.95 - 1.00)**	0.99 (0.97 - 1.00)**	1.00 (0.98 - 1.00)**	1.00 (1.00 - 1.00)**	1.00 (1.00 - 1.00)**	0.99 (0.96 - 1.00)*
Infant (>27 days, <2yrs)	0.92 (0.90 - 0.95)	0.92 (0.90 - 0.95)	0.94 (0.91 - 0.96)	0.94 (0.92 - 0.96)	0.96 (0.94 - 0.97)	0.97 (0.96 - 0.98)
Child[r] *(2yrs - 11yrs)*	0.92 (0.89 - 0.94)	0.93 (0.90 - 0.95)	0.93 (0.91 - 0.96)	0.93 (0.91 - 0.96)	0.93 (0.91 - 0.96)	0.95 (0.93 - 0.97)
Adolescent (12yrs - 18yrs)	0.95 (0.93 - 0.96)	0.95 (0.93 - 0.97)	0.96 (0.94 - 0.97)	0.96 (0.94 - 0.98)	0.96 (0.94 - 0.98)	0.97 (0.96 - 0.98)

The P-WIN XGB ensemble model's AUROC scores when restricted to individual demographic cohorts of the external validation dataset, at each prediction horizon. Cohorts containing fewer than 5 samples with each outcome are omitted.

[r]*Reference category.*

[*]*Statistically significant difference (Delong test P<0.05) compared with the reference group of the corresponding demographic field at the same prediction horizon.*

[**]*Statistically significant difference (Delong test P<0.001) compared with the reference group of the corresponding demographic field at the same prediction horizon*

Supplementary Table 8. *Counts of EHR variables used by each ensemble model.*

	XGB	RF	LASSO
Number of medication variables	335	372	339
Number of lab results variables	110	172	87
Number of vital signs variables	103	107	85
Number of demographic variables	2	2	2
Total number of variables	550	653	513

The number of EHR variables of each type used by the XGBoost (XGB), Random Forest (RF), and LASSO ensemble models. These counts only include variables with nonzero feature importance or model coefficient.

Supplementary Table 9. *Model variables used by any of the 12-, 24-, or 42-variable parsimonious models.*

Variable type	Variable name
Flowsheet	Arterial Mean Absolute Pressure (mm Hg)
Flowsheet	Diastolic Arterial Blood Pressure (mm Hg)
Flowsheet	End-Tidal CO2 (mm Hg)
Flowsheet	FiO2 (%)
Flowsheet	GCS Eye Opening (Age >= 2 years)
Flowsheet	Peak Inspiratory Pressure, Actual (cm H2O)
Flowsheet	State Behavioral Scale
Flowsheet	Systolic Arterial Blood Pressure (mm Hg)
Flowsheet	Transcutaneous CO2 (mm Hg)
Flowsheet	Urine Output (mL)
Flowsheet	Water/Blanket Temperature of Thermoregulation Device
Lab	Base Excess in Arterial Blood by Calculation (mmol/L)
Lab	Bicarbonate in Arterial Blood (mmol/L)
Lab	Bicarbonate in Venous Blood (mmol/L)
Lab	Calcium Ionized in Blood (mmol/L)
Lab	Carbon Dioxide (Partial Pressure) in Arterial Blood (mmHg)
Lab	Carbon Dioxide (Partial Pressure) in Venous Blood (mmHg)
Lab	Carbon Dioxide in Arterial Blood (mmol/L)
Lab	Carbon Dioxide in Venous Blood (mmol/L)
Lab	Chloride in Serum or Plasma (mmol/L)
Lab	Erythrocyte Distribution Width (%)
Lab	Erythrocyte Distribution Width (fL)
Lab	Fibrinogen in Plasma by Coagulation Assay (mg/dL)
Lab	Glucose in Blood (mg/dL)
Lab	Glucose in Serum or Plasma (mg/dl)
Lab	Hemoglobin in Blood (g/dL)
Lab	INR in Plasma by Coagulation Assay
Lab	Lactate in Arterial Blood (mmol/L)
Lab	Lactate in Venous Blood (mmol/L)
Lab	Magnesium in Serum or Plasma (mg/dL)
Lab	Oxygen in Arterial Blood (mmHg)
Lab	Oxygen in Venous Blood (mmHg)
Lab	Oxygen Saturation in Arterial Blood (%)
Lab	Oxygen Saturation in Venous Blood (%)
Lab	pH of Arterial Blood
Lab	pH of Venous Blood
Lab	Potassium in Blood (g/dl)

Lab	Prothrombin Time (sec)
Lab	Sodium in Blood (mmol/L)
Lab	Urea Nitrogen in Serum or Plasma (mg/dL)
Medication	Eye Washes/Lubricants
Medication	Ophthalmic Agents

Variables are ranked alphabetically within each variable type.

Supplementary Table 10. *Retrospective Alert Burden Study Results*

Percentage of events alerted	95%	90%	80%
Average of alerts-per-day across admissions (95% CI)[a]			
Overall	0.72 (0.70-0.73)	0.38 (0.37-0.40)	0.20 (0.19-0.21)
CDE admissions	1.50 (1.44-1.54)	1.18 (1.12-1.23)	0.86 (0.80-0.92)
Non-CDE admissions	0.70 (0.66-0.70)	0.35 (0.34-0.36)	0.17 (0.16-0.18)
Median of alerts-per-day across admissions (IQR)[b]			
Overall	0.59 (0.00-1.17)	0.00 (0.00-0.64)	0.00 (0.00-0.25)
CDE admissions	1.63 (1.29-1.82)	1.25 (0.86-1.51)	0.87 (0.48-1.20)
Non-CDE admissions	0.56 (0.00-1.09)	0.00 (0.00-0.58)	0.00 (0.00-0.16)
Median of time-to-event for true alerts (IQR), hrs	11.37 (6.05-17.87)	11.07 (5.40-17.66)	10.17 (5.20-17.30)
Number needed to alert (NNA) at the admission level (95% CI)[c]	17.20 (16.85-17.37)	12.64 (12.35-12.86)	9.29 (8.92-9.49)

Results of the P-WIN retrospective alert study on the external validation dataset. Predicted risk probabilities from the XGB ensemble model at time points every 1 hour for the duration of all PICU admissions were used to raise alerts with a 12-hour refractory period after alerts and a 24-hour censoring period after events. We provide metrics at three different alerting thresholds, for which 95%, 90%, and 80% of events were alerted within the preceding 24 hours, respectively. A true alert is an alert for which there is a future CDE within 24 hours.

The external validation set consists of 6,929 PICU admissions, 301 (4.3%) of which contain critical deterioration events (CDE) and 6,628 (95.7%) of which do not contain CDE.

[a]*Average across admissions of (Number of alerts during admission)/(Length of admission in days), with confidence intervals estimated using 2000 bootstrap replicates.*

[b]*Median (IQR) across admissions of (Number of alerts during admission)/(Length of admission in days)*

[c]*(Number of admissions with alerts)/(Number of admissions for which the first event was alerted in the prior 24 hrs)*

Supplementary Table 11. Alert Burden Comparison Between P-WIN and the PICU Warning Tool.

Prediction scheme	*PICU Warning Tool*	*P-WIN threshold A*	*P-WIN threshold B*
Percentage of events alerted (95% CI)	*37.6 (33.3-42.0)*	*66.3 (61.7-70.4)*	*38.9 (34.3-43.2)*
Average of alerts-per-day across admissions (95% CI) [a]	*0.10 (0.09-0.10)*	*0.10 (0.09-0.10)*	*0.03 (0.03-0.04)*
Number needed to alert (NNA) at the admission level (95% CI) [b]	*9.39 (8.31-10.58)*	*7.02 (6.50-7.63)*	*5.96 (5.23-6.92)*

Results of comparing metrics on the external validation dataset of two P-WIN alert thresholds versus PICU Warning Tool alerts. The two P-WIN thresholds were chosen to approximately match the percentage of events alerted or average alerts-per-day achieved by the PICU Warning Tool predictions, respectively. A CDE was considered to be alerted if there was an alert triggered in the preceding 24 hours. Predictions were generated every 1 hour, with a 12-hour refractory period after alerts and a 24-hour censoring period after events.

The external validation set consists of 6,929 PICU admissions, 301 (4.3%) of which contain critical deterioration events (CDE) and 6,628 (95.7%) of which do not contain CDE.

[a]*Average across admissions of (Number of alerts during admission)/(Length of admission in days), with confidence intervals estimated using 2000 bootstrap replicates.*

[b]*(Number of admissions with alerts)/(Number of admissions for which the first event was alerted in the prior 24 hrs).*

Supplementary Figures

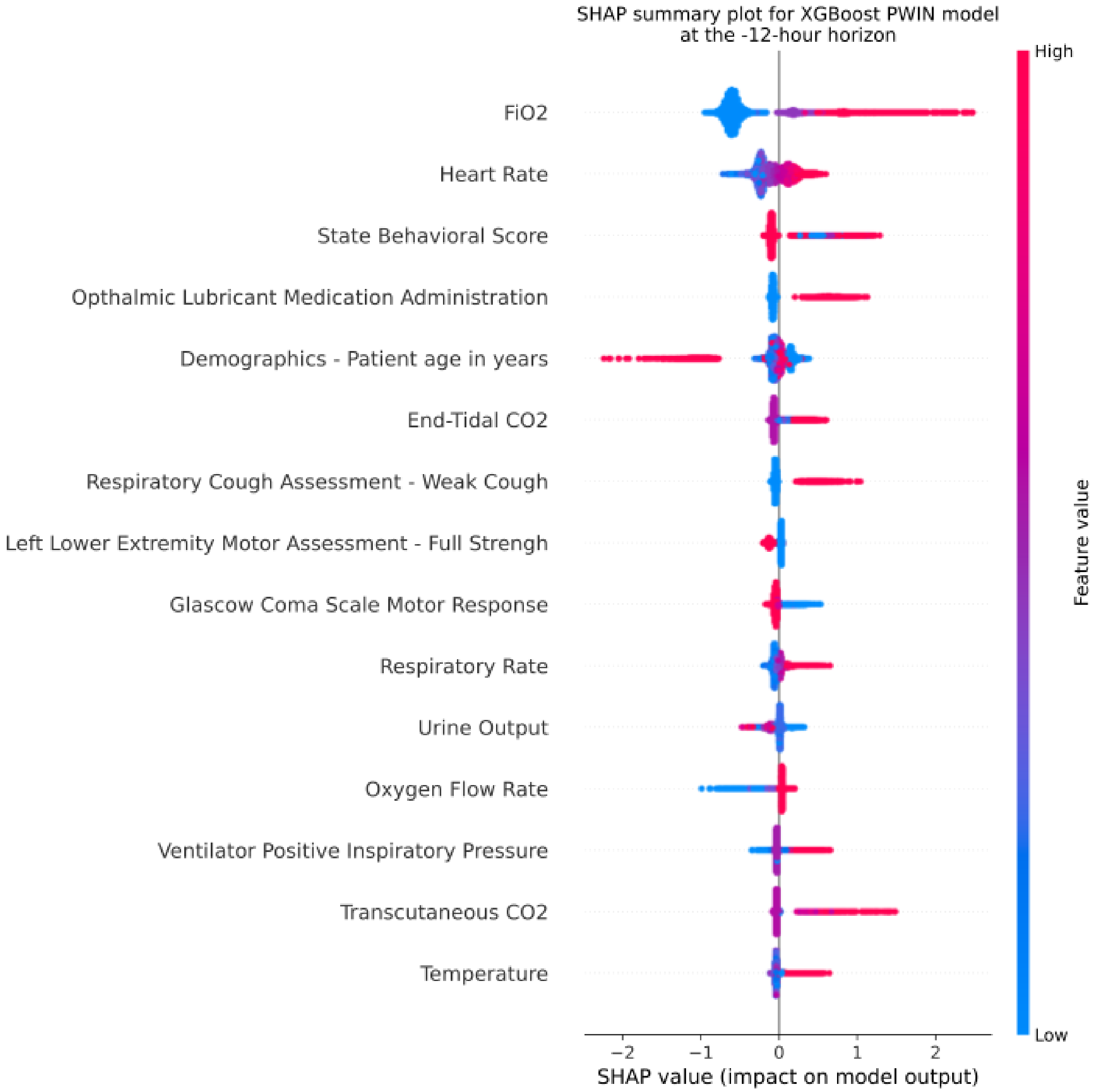

Supplementary Figure 1. *SHapley Additive exPlanations (SHAP) value results for the predictions of the XGBoost P-WIN 12-hour horizon model on the external validation dataset.*

We include the top 15 features, ranked by mean absolute SHAP value across this dataset.

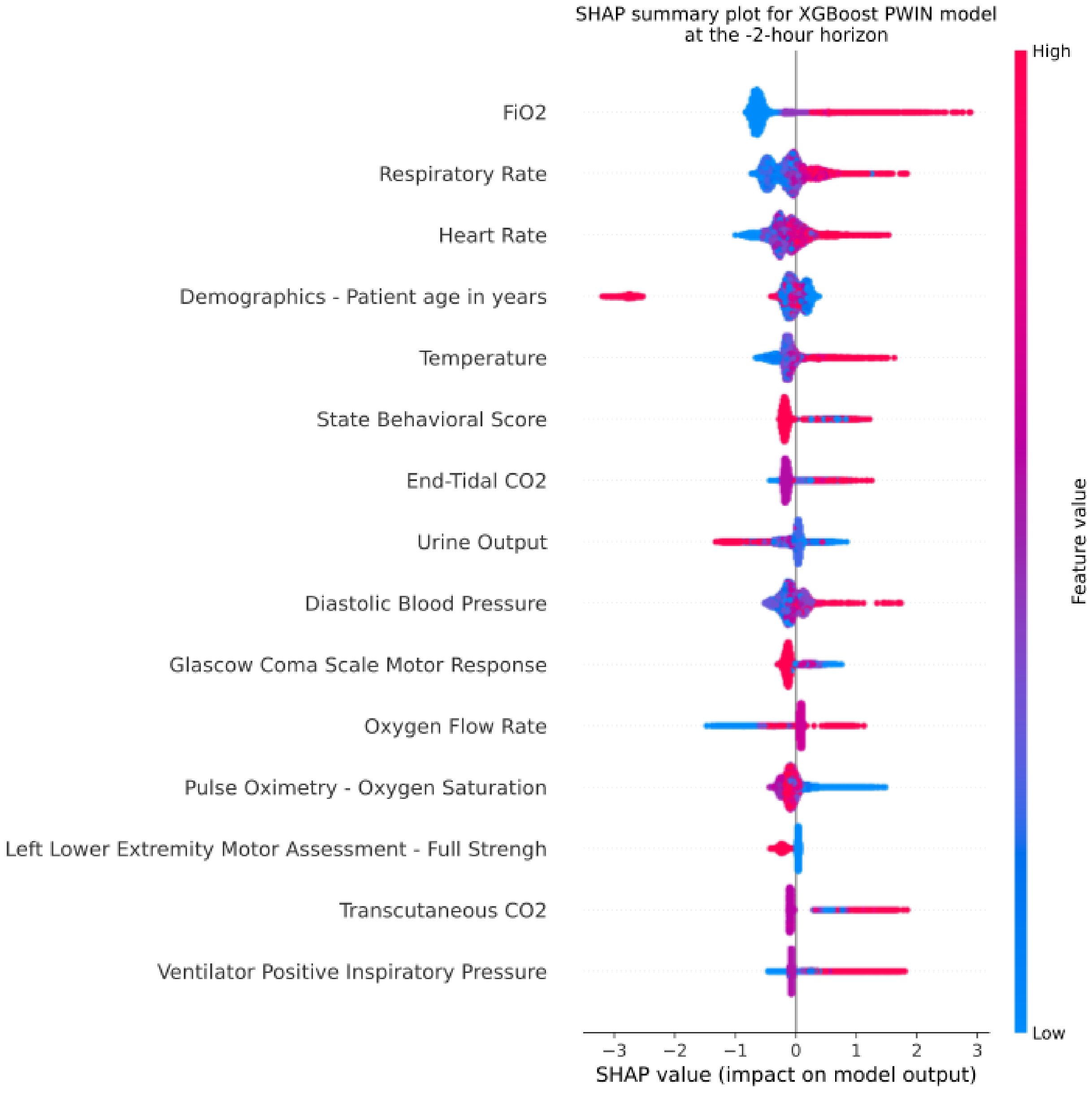

Supplementary Figure 2. *SHapley Additive exPlanations (SHAP) value results for the predictions of the XGBoost P-WIN 2-hour horizon model on the external validation dataset.*

We include the top 15 features, ranked by mean absolute SHAP value across this dataset.

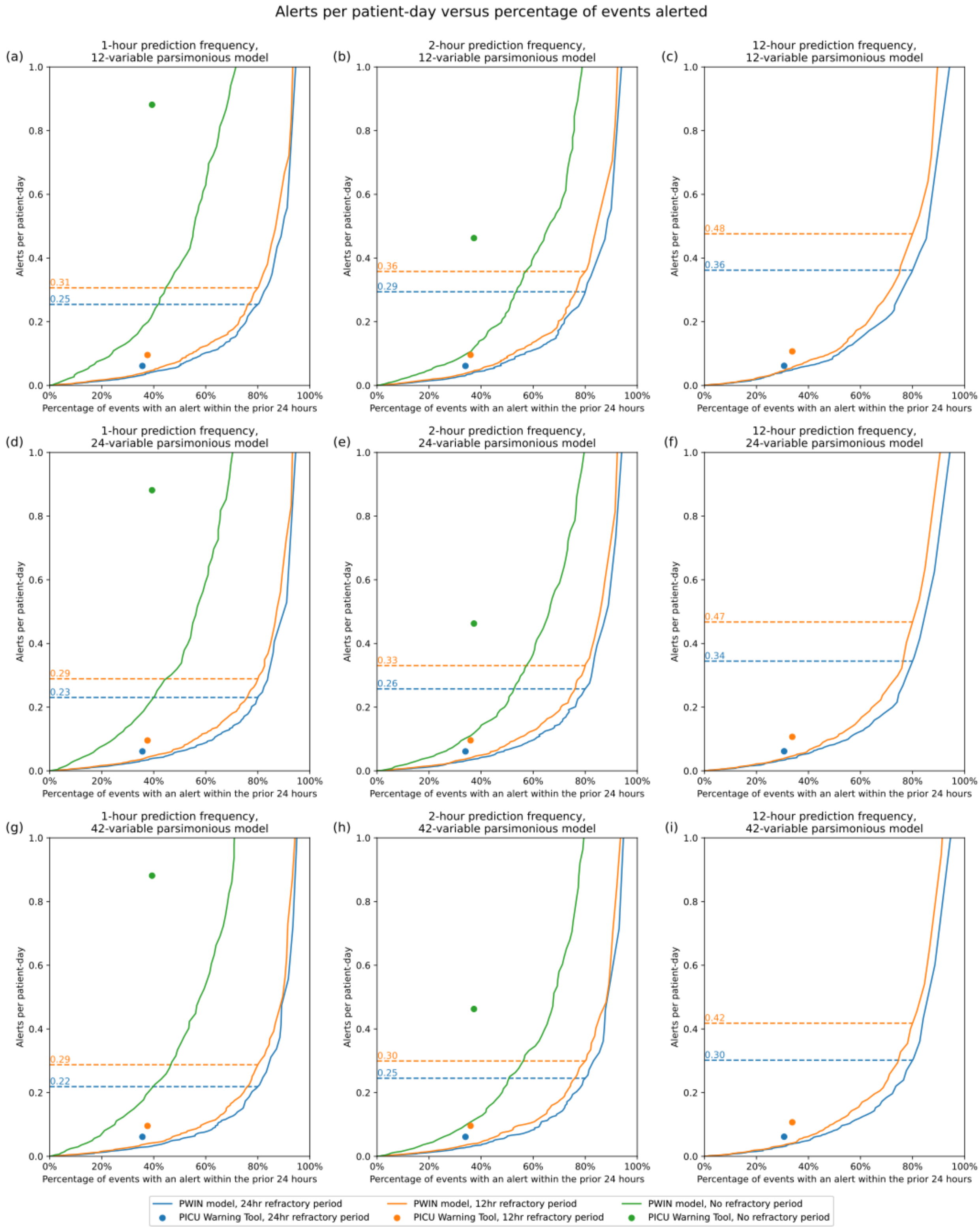

Supplementary Figure 3. *The average alerts per patient-day versus the percentage of CDEs for which an alert was raised, for various parsimonious models at all alert thresholds, using various prediction frequencies and refractory period lengths.*

Alerts were generated at a range of risk thresholds, and the resulting alert-per-patient-day burden was plotted against the percentage of CDEs for which an alert was raised in the preceding 24 hours. Parsimonious models used 12 variables (a-c), 24 variables (d-f), and 42 variables (g-i) respectively. Predictions were generated every (a,d,g) 1 hour, (b,e,h) 2 hours, or (c,f,i) 12 hours and using either no refractory period or 12-hour or 24-hour refractory periods. Note that only two curves appear on the right plot, as 12-hour refractory is equivalent to no refractory for 12-hour prediction frequency. Dashed lines indicate threshold values for which 90% of CDEs had alerts raised. Dots indicate the performance of the binary PICU Warning Tool alerts.

Tripod+AI Checklist

TRIPOD+AI

Version: 11-January-2024

Section/Topic	Item	Development / evaluation[1]	Checklist item	Reported on page
**TITLE**				
Title	1	D;E	Identify the study as developing or evaluating the performance of a multivariable prediction model, the target population, and the outcome to be predicted	1
**ABSTRACT**				
Abstract	2	D;E	See TRIPOD+AI for Abstracts checklist	3-4
**INTRODUCTION**				
Background	3a	D;E	Explain the healthcare context (including whether diagnostic or prognostic) and rationale for developing or evaluating the prediction model, including references to existing models	6-7
	3b	D;E	Describe the target population and the intended purpose of the prediction model in the context of the care pathway, including its intended users (e.g., healthcare professionals, patients, public)	6-7
	3c	D;E	Describe any known health inequalities between sociodemographic groups	
Objectives	4	D;E	Specify the study objectives, including whether the study describes the development or validation of a prediction model (or both)	6-7
**METHODS**				
Data	5a	D;E	Describe the sources of data separately for the development and evaluation datasets (e.g., randomised trial, cohort, routine care or registry data), the rationale for using these data, and representativeness of the data	9
	5b	D;E	Specify the dates of the collected participant data, including start and end of participant accrual; and, if applicable, end of follow-up	9
Participants	6a	D;E	Specify key elements of the study setting (e.g., primary care, secondary care, general population) including the number and location of centres	7-8
	6b	D;E	Describe the eligibility criteria for study participants	7-8
	6c	D;E	Give details of any treatments received, and how they were handled during model development or evaluation, if relevant	N/A
Data preparation	7	D;E	Describe any data pre-processing and quality checking, including whether this was similar across relevant sociodemographic groups	N/A
Outcome	8a	D;E	Clearly define the outcome that is being predicted and the time horizon, including how and when assessed, the rationale for choosing this outcome, and whether the method of outcome assessment is consistent across sociodemographic groups	8-9
	8b	D;E	If outcome assessment requires subjective interpretation, describe the qualifications and demographic characteristics of the outcome assessors	N/A
	8c	D;E	Report any actions to blind assessment of the outcome to be predicted	N/A
Predictors	9a	D	Describe the choice of initial predictors (e.g., literature, previous models, all available predictors) and any pre-selection of predictors before model building	9-10
	9b	D;E	Clearly define all predictors, including how and when they were measured (and any actions to blind assessment of predictors for the outcome and other predictors)	9-10, Supplement
	9c	D;E	If predictor measurement requires subjective interpretation, describe the qualifications and demographic characteristics of the predictor assessors	N/A
Sample size	10	D;E	Explain how the study size was arrived at (separately for development and evaluation), and justify that the study size was sufficient to answer the research question. Include details of any sample size calculation	8, Table 1
Missing data	11	D;E	Describe how missing data were handled. Provide reasons for omitting any data	Supplement
Analytical methods	12a	D	Describe how the data were used (e.g., for development and evaluation of model performance) in the analysis, including whether the data were partitioned, considering any sample size requirements	8
	12b	D	Depending on the type of model, describe how predictors were handled in the analyses (functional form, rescaling, transformation, or any standardisation).	Supplement
	12c	D	Specify the type of model, rationale[2], all model-building steps, including any hyperparameter tuning, and method for internal validation	10
	12d	D;E	Describe if and how any heterogeneity in estimates of model parameter values and model performance was handled and quantified across clusters (e.g., hospitals, countries). See TRIPOD-Cluster for additional considerations[3]	N/A
	12e	D;E	Specify all measures and plots used (and their rationale) to evaluate model performance (e.g., discrimination, calibration, clinical utility) and, if relevant, to compare multiple models	10-11
	12f	E	Describe any model updating (e.g., recalibration) arising from the model evaluation, either overall or for particular sociodemographic groups or settings	N/A
	12g	E	For model evaluation, describe how the model predictions were calculated (e.g., formula, code, object, application programming interface)	10-11
Class imbalance	13	D;E	If class imbalance methods were used, state why and how this was done, and any subsequent methods to recalibrate the model or the model predictions	N/A
Fairness	14	D;E	Describe any approaches that were used to address model fairness and their rationale	10
Model output	15	D	Specify the output of the prediction model (e.g., probabilities, classification). Provide details and rationale for any classification and how the thresholds were identified	10

[1] D=items relevant only to the development of a prediction model; E=items relating solely to the evaluation of a prediction model; D;E=items applicable to both the development and evaluation of a prediction model

[2] Separately for all model building approaches.

[3] TRIPOD-Cluster is a checklist of reporting recommendations for studies developing or validating models that explicitly account for clustering or explore heterogeneity in model performance (eg, at different hospitals or centres). Debray et al, BMJ 2023; 380: e071018 [DOI: 10.1136/bmj-2022-071018]

Page 1 of 2

Training versus evaluation	16	D;E	Identify any differences between the development and evaluation data in healthcare setting, eligibility criteria, outcome, and predictors	8, Table 1
Ethical approval	17	D;E	Name the institutional research board or ethics committee that approved the study and describe the participant-informed consent or the ethics committee waiver of informed consent	6
**OPEN SCIENCE**				
Funding	18a	D;E	Give the source of funding and the role of the funders for the present study	N/A
Conflicts of interest	18b	D;E	Declare any conflicts of interest and financial disclosures for all authors	N/A
Protocol	18c	D;E	Indicate where the study protocol can be accessed or state that a protocol was not prepared	N/A
Registration	18d	D;E	Provide registration information for the study, including register name and registration number, or state that the study was not registered	N/A
Data sharing	18e	D;E	Provide details of the availability of the study data	Submission content
Code sharing	18f	D;E	Provide details of the availability of the analytical code[4]	Submission content
**PATIENT & PUBLIC INVOLVEMENT**				
Patient & Public Involvement	19	D;E	Provide details of any patient and public involvement during the design, conduct, reporting, interpretation, or dissemination of the study or state no involvement.	N/A
**RESULTS**				
Participants	20a	D;E	Describe the flow of participants through the study, including the number of participants with and without the outcome and, if applicable, a summary of the follow-up time. A diagram may be helpful.	Figure 1
	20b	D;E	Report the characteristics overall and, where applicable, for each data source or setting, including the key dates, key predictors (including demographics), treatments received, sample size, number of outcome events, follow-up time, and amount of missing data. A table may be helpful. Report any differences across key demographic groups.	Table 1
	20c	E	For model evaluation, show a comparison with the development data of the distribution of important predictors (demographics, predictors, and outcome).	12, Table 1
Model development	21	D;E	Specify the number of participants and outcome events in each analysis (e.g., for model development, hyperparameter tuning, model evaluation)	12
Model specification	22	D	Provide details of the full prediction model (e.g., formula, code, object, application programming interface) to allow predictions in new individuals and to enable third-party evaluation and implementation, including any restrictions to access or re-use (e.g., freely available, proprietary)[5]	10
Model performance	23a	D;E	Report model performance estimates with confidence intervals, including for any key subgroups (e.g., sociodemographic). Consider plots to aid presentation.	11-13, Table 2, Figure 2
	23b	D;E	If examined, report results of any heterogeneity in model performance across clusters. See TRIPOD Cluster for additional details[3].	N/A
Model updating	24	E	Report the results from any model updating, including the updated model and subsequent performance	N/A
**DISCUSSION**				
Interpretation	25	D;E	Give an overall interpretation of the main results, including issues of fairness in the context of the objectives and previous studies	15-16
Limitations	26	D;E	Discuss any limitations of the study (such as a non-representative sample, sample size, overfitting, missing data) and their effects on any biases, statistical uncertainty, and generalizability	17
Usability of the model in the context of current care	27a	D	Describe how poor quality or unavailable input data (e.g., predictor values) should be assessed and handled when implementing the prediction model	N/A
	27b	D	Specify whether users will be required to interact in the handling of the input data or use of the model, and what level of expertise is required of users	N/A
	27c	D;E	Discuss any next steps for future research, with a specific view to applicability and generalizability of the model	18

From: Collins GS, Moons KGM, Dhiman P, et al. *BMJ* 2024;385:e078378. doi:10.1136/bmj-2023-078378

[4] This relates to the analysis code, for example, any data cleaning, feature engineering, model building, evaluation.
[5] This relates to the code to implement the model to get estimates of risk for a new individual.

References

1. Nelson SJ, Zeng K, Kilbourne J, Powell T, Moore R. Normalized names for clinical drugs: RxNorm at 6 years. J Am Med Inform Assoc. 2011 Jul-Aug;18(4):441-8. doi: 10.1136/amiajnl-2011-000116. Epub 2011 Apr 21. PMID: 21515544; PMCID: PMC3128404.
2. Carter JS, Brown SH, Bauer BA, Elkin PL, Erlbaum MS, Froehling DA, Lincoln MJ, Rosenbloom ST, Wahner-Roedler DL, Tuttle MS. Categorical information in pharmaceutical terminologies. AMIA Annu Symp Proc. 2006;2006:116-20. PMID: 17238314; PMCID: PMC1839555.
3. Forrey AW, McDonald CJ, DeMoor G, Huff SM, Leavelle D, Leland D, Fiers T, Charles L, Griffin B, Stalling F, Tullis A, Hutchins K, Baenziger J. Logical observation identifier names and codes (LOINC) database: a public use set of codes and names for electronic reporting of clinical laboratory test results. Clin Chem. 1996 Jan;42(1):81-90. PMID: 856539.
